# 529 Job Sharing: An Innovative Approach to Increasing Research Resources and Improving Staff Satisfaction

**DOI:** 10.1093/jbcr/irad045.126

**Published:** 2023-05-15

**Authors:** Karen Richey, Tiffany Hockenberry, Van Dobbe, Natalie Kesler, Suzanne Osborn, Claudia Islas, Kevin Foster

**Affiliations:** Arizona Burn Center Valleywise Health, Phoenix, Arizona; AZ Burn Center at Valleywise Health, Phoenix, Arizona; AZ Burn Center at Valleywise Health, Phoenix, Arizona; AZ Burn Center at Valleywise Health, Phoenix, Arizona; AZ Burn Center at Valleywise Health, Phoenix, Arizona; AZ Burn Center at Valleywise Health, Phoenix, Arizona; AZ Burn Center at Valleywise Health, Phoenix, Arizona; Arizona Burn Center Valleywise Health, Phoenix, Arizona; AZ Burn Center at Valleywise Health, Phoenix, Arizona; AZ Burn Center at Valleywise Health, Phoenix, Arizona; AZ Burn Center at Valleywise Health, Phoenix, Arizona; AZ Burn Center at Valleywise Health, Phoenix, Arizona; AZ Burn Center at Valleywise Health, Phoenix, Arizona; AZ Burn Center at Valleywise Health, Phoenix, Arizona; Arizona Burn Center Valleywise Health, Phoenix, Arizona; AZ Burn Center at Valleywise Health, Phoenix, Arizona; AZ Burn Center at Valleywise Health, Phoenix, Arizona; AZ Burn Center at Valleywise Health, Phoenix, Arizona; AZ Burn Center at Valleywise Health, Phoenix, Arizona; AZ Burn Center at Valleywise Health, Phoenix, Arizona; AZ Burn Center at Valleywise Health, Phoenix, Arizona; Arizona Burn Center Valleywise Health, Phoenix, Arizona; AZ Burn Center at Valleywise Health, Phoenix, Arizona; AZ Burn Center at Valleywise Health, Phoenix, Arizona; AZ Burn Center at Valleywise Health, Phoenix, Arizona; AZ Burn Center at Valleywise Health, Phoenix, Arizona; AZ Burn Center at Valleywise Health, Phoenix, Arizona; AZ Burn Center at Valleywise Health, Phoenix, Arizona; Arizona Burn Center Valleywise Health, Phoenix, Arizona; AZ Burn Center at Valleywise Health, Phoenix, Arizona; AZ Burn Center at Valleywise Health, Phoenix, Arizona; AZ Burn Center at Valleywise Health, Phoenix, Arizona; AZ Burn Center at Valleywise Health, Phoenix, Arizona; AZ Burn Center at Valleywise Health, Phoenix, Arizona; AZ Burn Center at Valleywise Health, Phoenix, Arizona; Arizona Burn Center Valleywise Health, Phoenix, Arizona; AZ Burn Center at Valleywise Health, Phoenix, Arizona; AZ Burn Center at Valleywise Health, Phoenix, Arizona; AZ Burn Center at Valleywise Health, Phoenix, Arizona; AZ Burn Center at Valleywise Health, Phoenix, Arizona; AZ Burn Center at Valleywise Health, Phoenix, Arizona; AZ Burn Center at Valleywise Health, Phoenix, Arizona; Arizona Burn Center Valleywise Health, Phoenix, Arizona; AZ Burn Center at Valleywise Health, Phoenix, Arizona; AZ Burn Center at Valleywise Health, Phoenix, Arizona; AZ Burn Center at Valleywise Health, Phoenix, Arizona; AZ Burn Center at Valleywise Health, Phoenix, Arizona; AZ Burn Center at Valleywise Health, Phoenix, Arizona; AZ Burn Center at Valleywise Health, Phoenix, Arizona

## Abstract

**Introduction:**

Pre-pandemic job openings for clinical research staff increased by nearly 10% a month nationwide yet was outpaced by an increase of greater than 12% in clinical trial activity. With the onset of the pandemic, the gulf between supply and demand widened in research. Simultaneously hospitals were stretched to capacity, staff suffered extreme burn-out and there was a dire need for additional bedside nurses. The pandemic shown a bright light on an age-old problem in nursing, whereas for a nurse to grow professionally, it often required leaving the bedside and giving up vital clinical skills, skills that were suddenly in great demand. The purpose of this project was to create a novel new job role, utilizing existing burn nursing staff to job share a research position, while maintaining their clinical expertise.

**Methods:**

Nurse leaders from both areas worked in concert with the burn center’s director of research, senior hospital administration, human resources and existing staff to develop a new job role. The job description incorporated the scope and standards for nursing practice in both areas into one document. Additionally, a second-level position was created which allowed bedside nurses to trial the new role without a formal commitment to the permanent position. Staff were scheduled to rotate every 2 weeks between the two areas. Two months following implementation, core research staff and job-sharing staff were polled for feedback on benefits and challenges.

**Results:**

Staff members (n=4) provided feedback independently from each other. A total of 41 comments were received, 27 benefit responses vs 14 challenges responses. Responses were grouped into themes and both benefits and challenges had 6 themes each. Benefit themes included: increased interdepartmental collaboration / trust (26%), professional growth (26%), supported learning (22%), enhanced screening for research patients (11%), unit-based resources (7%) and prevention of burnout (7%). Challenge themes included: scheduling (29%), variability in training opportunities (21%), role transitioning (21%), communication (14%), success personnel driven (7%), compensation (7%). Anecdotally, study investigators have expressed great satisfaction with the new position and there has been an increased interest expressed by other bedside staff in research involvement.

**Conclusions:**

The response to the new position has been overwhelmingly positive. Staff entered the trial period realizing that it may be “messy” at first but quickly adapted and have worked through many of the challenges. Based on this early success, we are exploring the viability of cross training additional staff.

**Applicability of Research to Practice:**

Cross training has been demonstrated to increase efficiencies, improve collaboration, trust and job satisfaction.

